# Encapsulation of Curcumin-Loaded Liposomes for Colonic Drug Delivery in a pH-Responsive Polymer Cluster Using a pH-Driven and Organic Solvent-Free Process

**DOI:** 10.3390/molecules23040739

**Published:** 2018-03-23

**Authors:** Vincenzo De Leo, Francesco Milano, Erminia Mancini, Roberto Comparelli, Livia Giotta, Angelo Nacci, Francesco Longobardi, Antonella Garbetta, Angela Agostiano, Lucia Catucci

**Affiliations:** 1 Department of Chemistry, University of Bari, Via Orabona 4, 70126 Bari, Italy; ermi.mancini@gmail.com (E.M.); angelo.nacci@uniba.it (A.N.); francesco.longobardi@uniba.it (F.L.); angela.agostiano@uniba.it (A.A.); 2 CNR-IPCF Institute for Physical and Chemical Processes, Bari unit, Via Orabona 4, 70126 Bari, Italy; francesco.milano@cnr.it (F.M.); r.comparelli@ba.ipcf.cnr.it (R.C.); 3 Department of Biological and Environmental Sciences and Technologies, University of Salento, SP Lecce-Monteroni, I-73100 Lecce, Italy; livia.giotta@unisalento.it; 4 CNR-ICCOM Institute of chemistry of organometallic compounds, Bari unit, Via E. Orabona, 4, 70126 Bari, Italy; 5 CNR-ISPA Institute of Sciences of Food Production, Via G. Amendola 122/O, 70125 Bari, Italy; Antonella.garbetta@ispa.cnr.it

**Keywords:** curcumin, nanoliposomes, Eudragit S100, colonic drug delivery, TEAC, pH jump method

## Abstract

The present study aimed to develop and optimize liposome formulation for the colonic delivery of biologically active compounds. A strategy to facilitate such targeting is to formulate liposomes with a polymer coating sensitive to the pH shifts in the gastrointestinal tract. To this end, liposomes encapsulating curcumin—chosen as the biologically active compound model—and coated with the pH-responsive polymer Eudragit S100 were prepared and characterized. Curcumin was encapsulated into small unilamellar vesicles (SUVs) by the micelle-to-vesicle transition method (MVT) in a simple and organic solvent-free way. Curcumin-loaded liposomes were coated with Eudragit S100 by a fast and easily scalable pH-driven method. The prepared liposomes were evaluated for size, surface morphology, entrapment efficiency, stability, in vitro drug release, and curcumin antioxidant activity. In particular, curcumin-loaded liposomes displayed size lower than 100 nm, encapsulation efficiency of 98%, high stability at both 4 °C and 25 °C, high in vitro antioxidant activity, and a cumulative release that was completed within 200 min. A good Eudragit S100 coating which did not alter the properties of the curcumin-loaded liposomes was obtained. The present work therefore provides a fast and solvent-free method to prepare pH-responsive polymer-coated liposomes for the colonic delivery of biologically active compounds.

## 1. Introduction

Curcumin is a polyphenol isolated from the rhizome of *Curcuma longa*, a plant originally from Southeast Asia, widely used as a spice and as a remedy in popular medicine. Numerous studies have confirmed over the years the potential beneficial effects of curcumin on human health due to its antioxidant, anti-inflammatory, antiamyloid, antidiabetic, anti-cystic fibrosis, and antimicrobial properties [1,2]. The interest in this molecule has undergone a tremendous increase since its anticarcinogenic activity also emerged, demonstrating its effectiveness through multiple mechanisms of action at various stages of the disease development [3,4]. However, curcumin shows a very poor bioavailability in vivo, due to its low aqueous solubility and instability, rapid metabolism, and clearance, that definitely limits its use both as a nutraceutical and as a drug [4]. Therefore, various strategies devoted to overcoming this limitation have been developed over the years, from the inclusion in suitable (nano)carriers to chemical modification and cocrystal synthesis [2,4,5,6,7,8,9]. A very popular and promising approach exploits the loading of curcumin into phospholipid-based liposomes. Liposomes are small vesicles arising from the spontaneous self-assembling of phospholipids dispersed in an aqueous medium. Lipid molecules organize themselves to form a double-layer structure very close to the natural cell membranes, enclosing an internal aqueous core. Liposomes are therefore biocompatible and biodegradable, and are widely used as delivery systems for stabilizing both hydrophilic and hydrophobic drugs and overcoming cellular and tissue barriers. Several drug-loaded liposomes are currently in clinical use [10].

Curcumin can be incorporated into liposomes with good yields and in a stable manner, with sustained release properties. Furthermore, compared with free curcumin, curcumin-loaded liposomes exhibited good stability against neutral and alkaline pH levels [1] that are found at the absorption site in the colonic region.

Colon-targeted drug delivery proves to be a profitable strategy for the treatment of both local and systemic diseases. Indeed, this region of the digestive system is characterized by near-neutral pH conditions, low enzymatic activity, low bile salt concentrations, and long residence time [11]. However, liposomes are not naturally suited to oral drug delivery due to their susceptibility to digestion [12].

Coupling the advantages of colonic and liposome drug delivery is a goal that can be achieved by coating vesicles with a suitable polymer layer, such as chitosan derivatives [6,13] or methacrylic acid copolymers [11,12,14]. In particular, Eudragit S100 is a polymer made of methacrylic acid and methacrylic acid methyl ester, with pH-dependent solubility and suitability for colonic drug delivery. Indeed, it allows the release of a cargo in the region of the gastrointestinal tract of pH > 7, that is, the large intestine or colon, where proteolytic enzymes and bile salt are low in concentration [15]. Previous works have shown the coverage of liposomes with Eudragit S100, exploiting the electrostatic interaction between the anionic polymer and cationic liposomes or using a double emulsion–solvent evaporation technique [11,12].

In this work, we encapsulated curcumin into small unilamellar vesicles (SUVs) or nanoliposomes by the micelle-to-vesicle transition (MVT) method in a simple, easy, effective, and organic solvent-free way [16]; and without introducing cationic components into the bilayer which may lead to toxic effects both in vitro and in vivo [17]. Afterwards, these curcumin-loaded liposomes were embedded into pH-responsive Eudragit S100 clusters. Differently from what has been proposed previously [12], we have achieved this encapsulation without the use of problematic organic solvents (dichloromethane, propanol, etc.) by a fast, simple, and easily scalable pH-driven method.

## 2. Results and Discussion

### 2.1. Liposome Preparation

**Figure 1:**
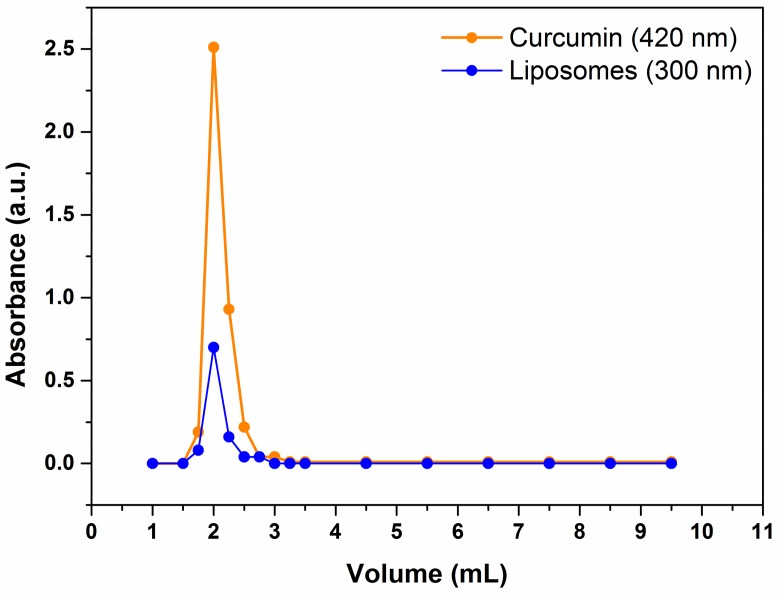
Elution profile of curcumin-loaded liposomes monitored by UV–vis spectra. Orange line: curcumin detected at 420 nm; blue line: liposome detected at 300 nm.

Curcumin-loaded liposomes were prepared by a suitable modification of the MVT method. This method does not require the use of organic solvents during the preparation and is carried out in aqueous buffers through an easy and rapid procedure. Starting from a suspension of mixed micelles made of detergent and lipid molecules, one can easily obtain small unilamellar vesicles (SUVs) with a good dimensional distribution by subtracting the detergent molecules from the microheterogeneous system [16]. In this work, mixed micelles were formed by sonication of a thin lipid–cholesterol–curcumin film added to a high-critical micellar concentration (CMC) detergent solution of sodium cholate. Due to their great hydrophobicity, curcumin molecules are expected to remain trapped within the lipid bilayer region of the mixed micelles during the procedure, similar to other hydrophobic molecules [18,19]. By means of size-exclusion chromatography (SEC), the detergent molecules were easily removed from the micelles. Closed vesicles of lipid and curcumin are formed when the detergent concentration in the aggregate phase becomes negligible, inducing the lipid reorganization [20]. Figure 1 shows the elution profile obtained during SEC, namely the variation of the absorbance at 420 nm (corrected for the scattering; orange line) corresponding to the absorption peak of curcumin, and the variation of the scattering recorded at 300 nm (blue line) corresponding to the liposome light diffusion, as a function of the eluate volume. Liposomes emerged from the column after a void volume of 1.5 mL, in a total volume of 1 mL, as expected. Figure 1 shows that curcumin coelutes with liposomes, since the maximum curcumin absorption coincides with the maximum liposome scattering. Therefore, curcumin remains trapped in the bilayer, as demonstrated previously for other methods of liposome preparation [18]. The absence of subsequent peaks in the curcumin elution profile indicates that curcumin does not escape in the free form from the column and does not form polymolecular aggregates with the high-CMC detergent [21].

**Figure 2:**
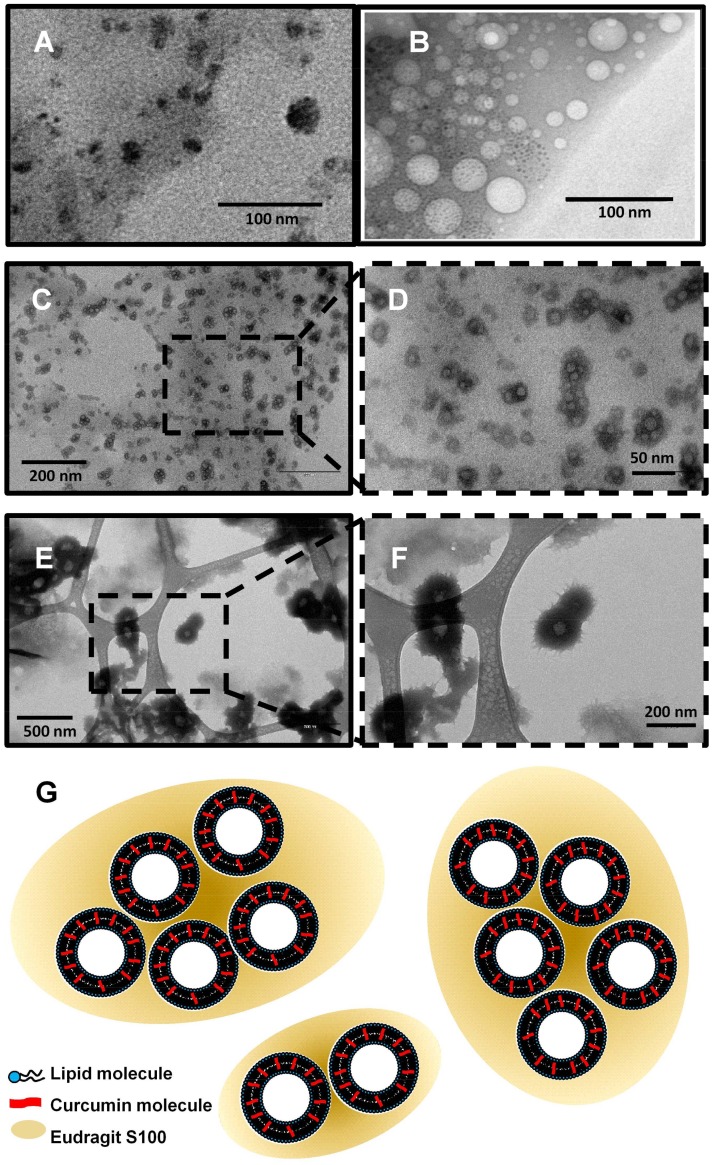
TEM images of control Eudragit S100 particles (**A**) and liposomes (**B**); and curcumin-loaded liposomes at 5 × 10^−5^ M (**C**,**D**) and 1 × 10^−3^ M (**E**,**F**) concentrations, coated with Eudragit S100 polymer. (**G**): schematic representation of liposomes coated with Eudragit S100 obtained with the pH jump method.

The encapsulation efficiency (EE%) of curcumin-loaded liposomes was found to be dependent on the curcumin/lipid ratio, resulting almost quantitatively for a maximum curcumin content of 0.74 µg/mg of total lipids and progressively decreasing at higher ratios (Table 1). Over the saturation condition of the lipid phase, the surplus of curcumin appears to be removed from the liposomes’ bilayer during the gel-permeation process. This behavior is confirmed by the change of color of the SEC resin that turns reddish, indicating the formation of a curcumin precipitate inside the pores of the gel. However, while decreasing the encapsulation yield at high curcumin concentrations, the absolute amount of trapped antioxidant increases, probably due to a mass effect that causes a rearrangement of the lipid bilayer. In fact, Transmission Electron Microscopy (TEM) images show an increase of the liposome bilayer thickness occurring at high concentrations of curcumin (Figure 2E,F for curcumin at 1 mM). Table 1 shows how the average size of the vesicles obtained is always well below 100 nm; also in the case of the liposomes at the highest curcumin concentration achieved. As the cellular uptake of liposomes significantly depends on their size, the MVT method is confirmed as suitable for obtaining liposomes with physicochemical characteristics which may improve their colon-targeted drug delivery properties.

### 2.2. Liposome Stability

**Figure 3:**
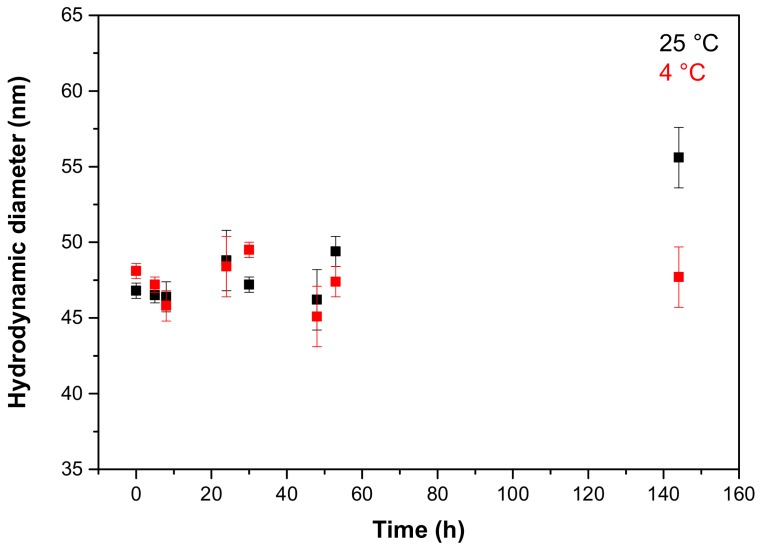
Time stability of curcumin-loaded liposomes at 25 °C and 4 °C.

The stability of the curcumin-loaded liposomes was studied by monitoring their size at 25 °C and 4 °C (Figure 3). The results showed that both at room temperature and at 4 °C the vesicles were essentially stable for six days. The load of curcumin therefore does not significantly affect the stability of the vesicles, which undergo only small variations in their size during the observed period.

### 2.3. Curcumin Release

**Figure 4:**
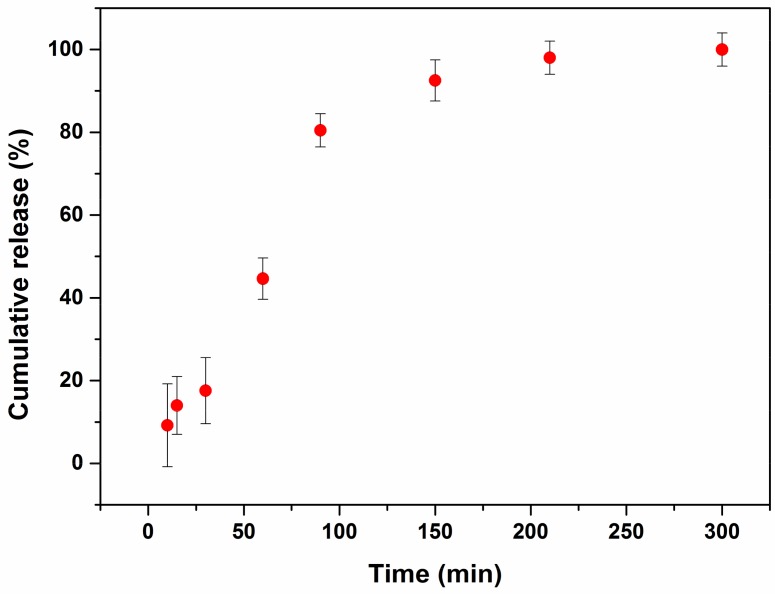
Release time course of liposomes loaded with 10^−5^ M curcumin. The error bars represent the mean and standard deviations of the experiments (*n* = 3).

We have studied the release process of curcumin-loaded liposomes in order to evaluate their potential as a curcumin delivery system. Figure 4 shows the results obtained for liposomes with 10^−5^ M of curcumin. Similarly to a previous report, we studied curcumin release to oleic acid as a model of the cell membrane environment. Curcumin indeed is a very hydrophobic compound, with a strong tendency to reside in the lipid bilayer rather than to be released to aqueous media [6]. We found that at the beginning the release is slow and gradual and then undergoes a slight acceleration. The release is complete within 200 min.

### 2.4. Liposome Coating with Eudragit S100

**Figure 5:**
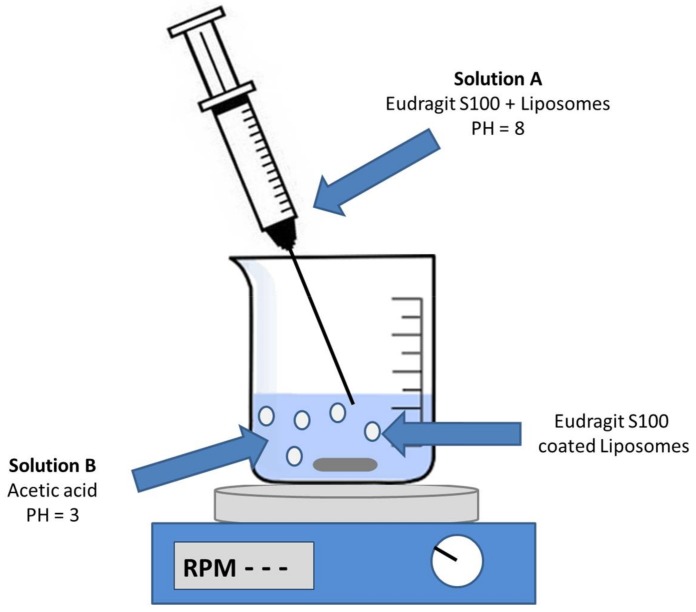
Schematic representation of liposomal coating with Eudragit S100 by the pH jump method.

The polymer-coated liposome production in this study was based on a pH-driven technique entirely carried out in water. Preliminary studies were carried out by dissolving the Eudragit S100 in buffer at pH 8.0, and carrying out a nanoprecipitation in a 0.25% acetic acid solution at pH 3.0 through the use of a syringe and under magnetic stirring. The rapid pH jump induced the precipitation of the polymer as nanometer-scale aggregates, detected by electron microscopy (Figure 2A). By adding the liposomes (TEM image in Figure 2B) to the Eudragit S100 alkaline solution (Solution A, Figure 5) and injecting it into the acid solution (Solution B, Figure 5), the polymer precipitation around the vesicles occurred. TEM images showed that the polymer does not include the individual liposomes, but rather a series of liposomes forming clusters of variable shape and size (Figure 2C,D, and cartoon). It is important to underline that the images did not reveal the segregation phenomena of vesicles and separate polymer aggregates. High speeds of injection and stirring and the mixing of the two solutions A and B in the ratio 1:9 were critical parameters for the control of the cluster dimensions (see details in Materials and Methods). Measuring the size of the liposomes covered by Eudragit S100 by Dinamic Light Scattering (DLS) was hindered by the irregular shape of the aggregates—far from the spherical symmetry required for this type of measurement—and their high polydispersity. However, TEM images showed that the dimensions of Eudragit S100-coated liposomes remained in a submicrometric scale. The TEM images, however, did not allow us to determine whether the polymer forms a continuous solid layer around the vesicles; therefore, appropriate experiments have been carried out as described below.

### 2.5. Reaction Center (RC) Assay

The effective coverage of liposomes by Eudragit S100 has been tested by exploiting the spectroscopic signals arising from the photosynthetic reaction centers (RCs) of the bacterium *Rhodobacter sphaeroides*. The RC is an integral membrane protein that, upon light absorption, generates a charge-separated state within its cofactors [22]. A special pair of bacteriochlorophylls acts as the primary electron donor (D) and a quinone molecule acts as the electron acceptor. The charge-separated state formation is accompanied by a marked decrease of the protein absorbance at 865 nm, followed by a charge recombination reaction that occurs in the time scale of seconds. In the presence of electron donors to the special pair, the RCs can perform more turnovers upon repetitive flash excitations. In living cells, RCs are vectorially oriented in the plasmatic membrane, with D facing towards an extracytoplasmic compartment called the periplasm, and the quinone binding site towards the cytoplasm. Cytochrome c_2_, solubilized in the periplasm, donates electrons to the oxidized primary donor, D^+^. In vitro, if the RCs are reconstituted in liposomes and reduced mitochondrial cytochrome c^2+^ (cyt c^2+^), which does not penetrate across the lipid layer, is added as an external electron donor, the amplitude of the absorbance change at 865 nm (ΔA_865_) can be used to study the D accessibility from the aqueous external phase. In fact, in the RCs with D faced towards the liposome’s inner aqueous core, D^+^ can be reduced only by charge recombination with the quinone; while the RCs of opposite orientation can also undergo microsecond time scale D^+^ reduction by cyt c^2+^. A comparison of the amplitude of ΔA_865_ before and after the addition of cyt c^2+^ shows that the typical orientation of liposome-reconstituted RC is close to 50% inside out, as the signal is halved after cyt c^2+^ addition.

**Figure 6:**
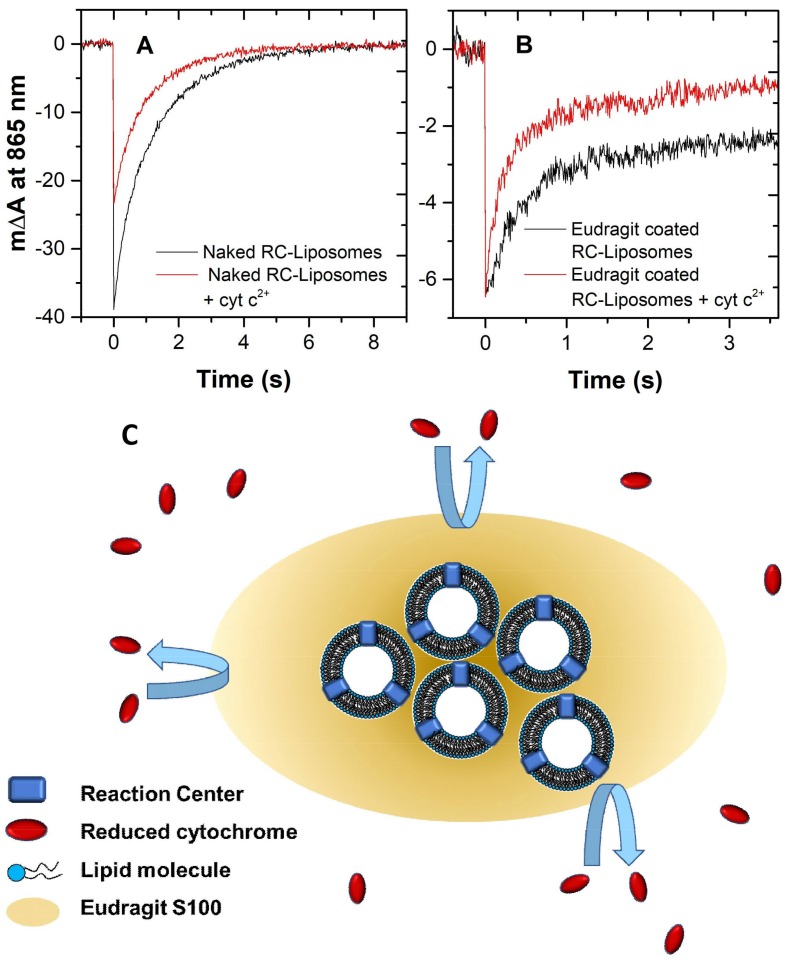
Flash-induced absorbance change of photosynthetic reaction centers (RC); (**A**): in naked liposomes before (red trace) and after (black trace) the addition of reduced cyt c; (**B**): in Eudragit-coated liposomes before (red trace) and after (black trace) the addition of reduced cyt c. (**C**): schematic representation of a cluster of RC-containing liposomes made inaccessible to the externally added cytochrome due to the Eudragit covering.

This assay can be exploited to study the Eudragit S100 coverage of liposomes [23]. In the case of successful coverage, in fact, the cyt c^2+^ is no longer able to interact with the RC with D facing outwards, and the signal before and after the cyt c^2+^ addition should remain the same. In our case, we have prepared RC-containing liposomes made of LS100 with a final protein concentration of 0.5 µM and a lipid/protein ratio of 1000:1 in 50 mM phosphate buffer and 100 mM KCl at pH 7.0. The ΔA_865_ time course upon flashing light excitation is shown in Figure 6A (black trace), and compared to the signal after the addition of 20 µM cyt c^2+^ from a 1 mM stock solution (red trace). The halving of the signal indicates, as expected, a random orientation of RCs in our preparation. The Eudragit S100 covering has been performed as explained in Section 2.4. The ΔA_865_ of the covered sample is shown in Figure 6B (black trace), where the initial amplitude is reduced proportionally with the dilution factor. Upon the addition of cyt c^2+^, however, the amplitude of the signal remains unchanged (red trace), featuring only a slightly faster decay in the dark, indicating the successful covering of liposomes by Eudragit S100.

Some authors suggest that the structure of the Eudragit S100 layer around the vesicles may be porous in nature, thus allowing access to small molecules such as bile salts, with deleterious consequences on the structure of liposomes trapped within the polymer cluster [11]. For this reason, we have subjected the liposomes covered by Eudragit S100 to the action of 10 mM sodium cholate solution, a bile salt capable of breaking up the liposomes and transforming them into mixed micelles. After two hours of incubation, the supernatant was separated and analyzed by UV–vis spectroscopy. No release of curcumin was highlighted. Furthermore, after dissolving the polymeric shell of Eudragit-coated liposomes at pH 8.0 and releasing the liposomes, the measurements performed with DLS did not show significant variations in the diameter of the vesicles (data not shown). Therefore, we exclude the access of the sodium cholate into the polymeric structures, with the structures realized by the pH jump method.

### 2.6. Antioxidant Activity

**Figure 7:**
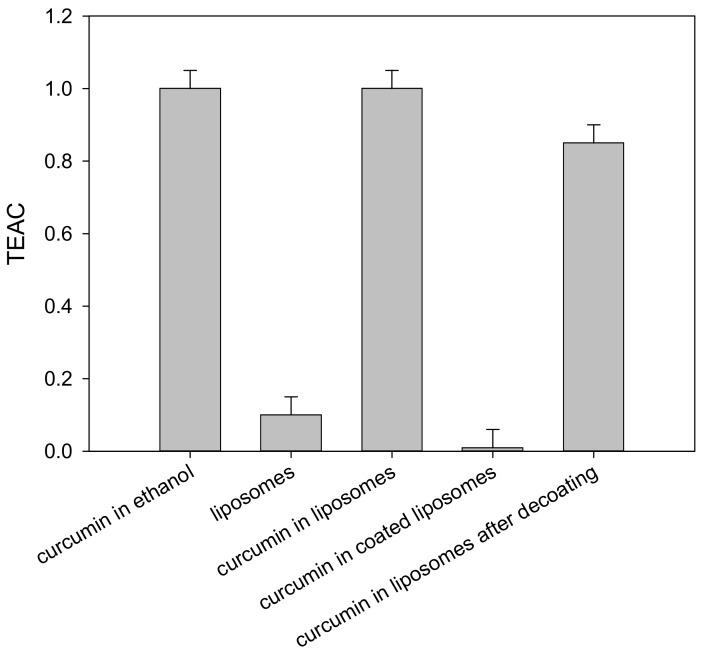
Trolox equivalent antioxidant capacity (TEAC) of curcumin in the various environments tested.

The antioxidant activity of curcumin in liposomes was also measured and compared with that of free curcumin dissolved in ethanol (Figure 7). Curcumin is a phenolic antioxidant which scavenges free radicals, acting as an H-atom donor from its phenolic groups [24], and the evaluation of its antioxidant activity was performed using the well-known 2,2′-azino-bis(3-ethylbenzothiazoline-6-sulphonic acid) (ABTS) decolorization assay (see Section 3.8).

As depicted in Figure 7, the ABTS**^•^**^+^ scavenging ability of curcumin-loaded liposomes was very close to that of free curcumin in ethanol, indicating that the liposome encapsulation process does not affect curcumin’s antioxidant properties. Furthermore, empty (curcumin-free) liposomes exhibited only a very weak activity attributable to phosphatidylcholine (Lipoid S100) used for the assembly of vesicles; phosphatidylcholine is the main component of low-melting point lecithin, which has been demonstrated to act as an oxidative stabilizer in emulsions [25].

In addition, the antioxidant activity of curcumin in Eudragit-coated liposomes was also measured, and compared with that of curcumin in liposomes after dissolution of the polymer shell in alkaline conditions (Figure 7). The experiments showed that curcumin-loaded liposomes covered by Eudragit S100 did not show any appreciable activity, thus confirming a good coverage of vesicles and the shielding of curcumin from the aqueous environment. Moreover, the almost complete recovery of antioxidant activity recorded for the same sample after Eudragit S100 dissolution demonstrates that the polymer coverage does not alter the properties of curcumin-loaded liposomes.

### 2.7. In Vitro Cytotoxicity

**Figure 8:**
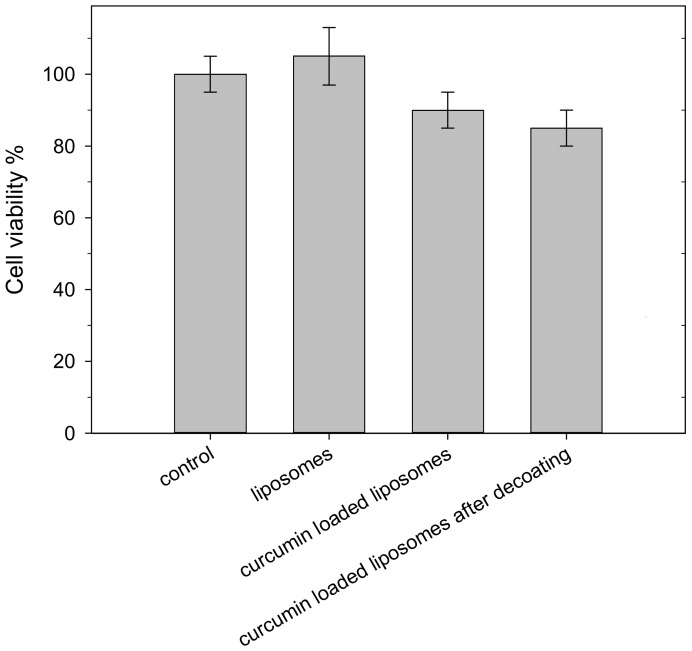
Cell viability of liposome formulations. A Caco-2 human colon cell line was incubated with liposomes, curcumin-loaded liposomes, and curcumin-loaded liposomes after decoating for 24 h. Control indicates untreated cells. Data, assessed as percentage of control samples, are expressed as mean ± SD (*n* = 12).

Preliminary experiments were carried out to assess the possible in vitro cytotoxicity of our liposomal formulations on the Caco-2 human colon cell line. As shown in Figure 8, no significant reduction of cell viability was observed after exposure to the liposomal suspension with/without curcumin or the liposomal suspension after decoating (but without Eudragit removal), confirming the safety of these samples for the intestinal cell line.

## 3. Materials and Methods

### 3.1. Materials

All chemicals were purchased at the highest purity available and were used without further purification. Ethanol, Sephadex G-50 medium, cholesterol, curcumin, oleic acid, the reagent grade salts for the 50 mM K–phosphate and 100 mM KCl (pH 7.0) buffer solutions, 2,20-azinobis(3-ethylbenzothiazoline-6-sulfonic acid) diammonium salt (ABTS), 6-hydroxy-2,5,7,8-tetramethylchroman-2-carboxylic acid (Trolox), and sodium cholate (SC) were purchased from Sigma-Aldrich. Lipoid S100 (LS100) was from Lipoid (Lipoid GmbH, Ludwigshafen, Germany). According to the manufacturer’s information, LS100 is constituted of approximately 100% soybean phosphatidylcholine. Eudragit S100 was a kind gift of Evonik (Evonik Industries AG, Darmstadt, Germany). Sterile filters of cellulose acetate (0.2 µm) were from Advantec. All aqueous solutions were prepared by using water obtained from a Milli-Q gradient A-10 system (Millipore, Burlington, MA, USA, 18.2 MΩ cm, organic carbon content ≤ 4 μg/L).

### 3.2. Liposome Preparation

Liposomes were prepared by the MVT method, consisting of detergent removal from phospholipid/detergent mixed micelles to induce liposome formation, as previously described [26]. In brief, an adequate amount of lipids (usually 10 mg of Lipoid S100/cholesterol 10:1 *w*/*w*) was dissolved in ethanol and dried with a gentle nitrogen flux to form a homogeneous film on the walls of a conical glass tube. Solvent removal was completed under vacuum conditions (24 h) by a no-oil pump operating at 1 mbar. After that, 0.5 mL of 4% SC in 50 mM K–phosphate/100 mM KCl (pH 7.0) was added to the dry lipid film and then sonicated (20 shots with a Branson Sonicator 250) to form a clear, translucent mixed micelle solution. The latter was loaded onto a glass column (20 × 1 cm) packed with G-50 Sephadex medium equilibrated with 50 mM K–phosphate/100 mM KCl (pH 7.0) for detergent removal by size exclusion chromatography (SEC). Liposomes were eluted in a 1 mL fraction after a void volume of about 1.5 mL. For curcumin-loaded liposomes, an appropriate amount of antioxidant was added to the lipid blend.

### 3.3. Preparation of the ES100-Coated Liposomes

SUVs were encapsulated within Eudragit S100 using a pH-driven method. Eudragit S100 was dissolved in a 100-mM alkaline phosphate buffer, pH 8.0, to produce a 0.25% solution (*w*/*w*) (Alkaline Solution). Equal volumes of a 5 mg/mL suspension of liposomes and Alkaline Solution were mixed for several minutes, to form solution A. Solution A was placed in a syringe and quickly injected into a 0.25% (*v*/*v*) solution of glacial acetic acid in water, pH ≈ 3.0 (solution B) under magnetic stirring (500 rpm). Solution A and solution B were mixed in a 1:9 dilution ratio. Eudragit S100-coated liposomes were then harvested by centrifugation and washed with distilled water to remove any excess of reagents.

### 3.4. Vesicle Size Assessment

Liposome preparations’ hydrodynamic radius at 25 °C was obtained by DLS (Nanoparticle Size Analyzer, Horiba Jobin Yvonne LB-550, Horiba Jobin Yvon SRL, Opera Milano, Italy), with 100 samplings per measurement. The power spectrum (PS) obtained by fast Fourier transformation of the light intensity fluctuations was converted into particle size distribution by comparing it, using the Twomey method [27], with the calculated frequency distribution based on the Brownian movement for particles of different size.

### 3.5. Stability of Curcumin Loaded Liposomes

The stability of curcumin-loaded liposome formulations was investigated by their incubation at two different temperatures: 25 °C (100 rpm) and 4 °C (with no stirring). The particle size was measured at different time intervals after a 1:3 dilution in double-distilled water, by DLS. Each experiment was performed in triplicate.

### 3.6. Ultraviolet-Visible Spectroscopy

Ultraviolet and visible spectra of solutions and suspensions were acquired by means of a Cary 5000 (Agilent Technologies, Santa Clara, CA, USA) ultraviolet-visible double-beam spectrophotometer. The resolution was set to 1 nm, and Hellma quartz cuvettes of 1 cm path length and suitable capacity were employed.

### 3.7. Curcumin Release from Liposomes

The experiments were carried out as previously described [6]. In brief, curcumin-loaded liposome suspension (1 mL, pH 7.5) was added to 2 mL of oleic acid and incubated at 25 °C with steady agitation (50 rpm). After defined periods of time, the sample was taken out, the oleic phase (upper phase) was separated by stratification, and the absorbance was measured (λ = 420 nm, absorption maximum of curcumin in oleic acid). The experiments were done in triplicate. Mean values and calculated SD were presented as error bars in the graph.

### 3.8. Antioxidant Activity

The evaluation of antioxidant activity was performed using the well-known ABTS decolorization assay [28], suitably adapted for the analysis of liposome-embedded antioxidant samples [18]. The assay is based on the preventive formation of the radical cation ABTS^•+^, and on the subsequent evaluation of its neutralization by the antioxidant compound(s) present in the sample. The reaction is easily followed spectrophotometrically, since ABTS^•+^ is a colored species, unlike the neutral form (hence the name of decolorization assay). The antioxidant-induced absorbance changes were recorded at 734 nm. Trolox was used as the standard antioxidant for the construction of suitable calibration curves. The antioxidant power was expressed as the Trolox equivalent antioxidant capacity (TEAC).

### 3.9. Transmission Electron Microscopy

The liposome morphology was assessed by transmission electron microscopy (TEM). Micrographs were recorded using a Jeol JEM-1011 microscope working at an accelerating voltage of 100 kV and acquired by an Olympus Quemesa Camera (11 Mpx). Samples were prepared by dropping the liposome aqueous suspension on a 400-mesh amorphous carbon-coated Cu grid and letting the solvent evaporate. The samples were stable under the electron beam and did not degrade within the typical observation times. Positive-staining TEM experiments were performed by dipping the grid, after the sample deposition, in a 2% (*w*/*v*) phosphotungstic acid solution for 30 s. The staining agent excess was removed from the grid by rinsing with ultrapure water (dipping the grid in ultrapure water three times for 10 s). The sample on the grid was left to dry overnight and finally stored in a vacuum chamber until analysis.

### 3.10. Cytochrome/RC Assay for Liposome Coverage Assessment

RC-containing liposomes made of LS100 with a final protein concentration of 0.5 µM and lipid/protein ratio of 1000:1 in 50 mM phosphate buffer, 100 mM KCl, pH 7.0 have been prepared using the MVT method, as reported elsewhere [22]. The amplitude of the photoinduced absorbance change has been detected at 865 nm with a kinetic spectrophotometer of local design, as described elsewhere [29]. The fraction of RCs with dimers facing outwards was determined by recording the same signal after the addition of 20 µM cyt c^2+^ from a 1 mM stock solution. These measurements were finally performed with Eudragit-covered liposomes.

### 3.11. Cell Cultures and Cytotoxicity

The human colon carcinoma cell line Caco-2, clone TC7, kindly supplied by Dr. Rousset, (INSERM U505, Paris, France), was grown in 25 cm^2^ flasks at a starting density of 250,000 cells/mL in Dulbecco’s Modified Eagle’s Medium with 4.5 g/L glucose, supplemented with 10% of Fetal. Bovine Serum (FBS), 1% of L-glutamine, 1% of anantibiotic and antimycotic solution, and 1% of a nonessential amino acid solution. Cell density and viability were assessed with a Scepter 2.0 automated handheld cell counter (Merk Millipore). To test the in vitro cytotoxicity of liposomal systems, in brief, the Caco-2 human colon cell line was seeded at a cell density of 100,000 cells/mL in 96-well plates, exposed to 100 µL of liposomes (5 mg/mL total lipids, 10 µM curcumin) dispersed in culture medium, and incubated for 24 h. After the incubation time, the influence of liposomal systems on cell viability was assessed by MTT test following the protocol described by Minervini et al [30].

## 4. Conclusions

Lipid-based colon-targeted delivery systems require coating to avoid cargo release in the stomach and small intestine. Within this context, nanoliposomes coated with pH-sensitive polymers have emerged as an attractive strategy to reduce this undesirable effect. Indeed, compared to other nanostructured drug delivery systems developed for colonic delivery, such as solid lipid nanoparticles and polymeric nanoparticles [31], liposomes have the undoubted advantage of being able to trap both hydrophilic and hydrophobic components (in the bilayer and in the inner aqueous core, respectively). Moreover, liposomes can easily be decorated with different molecules such as hydrophilic polymers, enzymes, and antibodies in order to stabilize the vesicles and promote their active delivery, in addition to a passive accumulation in the inflamed colonic region [32]. Furthermore, the surface charge can be appropriately modulated by choosing the components of the lipid blend. In this work, nanovesicles coated with the pH-responsive polymer Eudragit S100 were prepared using a pH-driven and organic solvent-free process. In particular, the MVT method was optimized for the preparation of liposome-encapsulated curcumin, chosen as the biologically active compound model with known antioxidant properties. The method allowed us to obtain nanoliposomes with an average hydrodynamic diameter ranging from 35 to 70 nm, depending on the amount of incorporated curcumin. The maximum EE % obtained was around 98%. The prepared systems were very stable, showing a low tendency to aggregate both at 4 °C and 25 °C. Also, the antioxidant and release properties have proved excellent, with a TEAC comparable with that of free curcumin and a cumulative release complete after 200 min. The liposomes were coated with the pH-sensitive polymer Eudragit S100 using a fast and easily scalable pH-driven method, which proved to be effective as confirmed by the “reaction center assay” and morphological analysis. TEM images, in fact, revealed that the Eudragit S100 coating allowed us to obtain a structure in which the polymer includes several liposomes forming clusters of variable shape and size. Such a coating was effective in protecting the liposomes, which retained high curcumin antioxidant activity after dissolution of the polymer shell in alkaline conditions.

These results demonstrate that the adopted strategy used is suitable for the development of lipid systems for colonic delivery of hydrophobic bioactive molecules such as curcumin. In particular, the MVT method coupled to the proposed pH jump method for the covering of liposomes with pH-sensitive polymers should expand the use of molecules characterized by a low colonic absorption and a high tendency to degradation, both as nutraceuticals and as drugs for the treatment of both local and systemic diseases.

## Figures and Tables

**Table 1:** Curcumin encapsulation efficiency (EE %) and mean diameter of liposomes as a function of curcumin loading.

Sample	Curcumin (M)	Curcumin/Lipids Mass Ratio (µg/mg)	EE (%)	Mean Diameter (nm)
Empty	-	-	-	42 ± 7
1	10^−5^	0.74	98 ± 4	36 ± 6
2	5 × 10^−5^	3.68	42 ± 3	40 ± 7
3	1.5 × 10^−4^	11.0	35 ± 2	47 ± 9
4	3 × 10^−4^	22.1	25 ± 2	56 ± 12
5	7 × 10^−4^	51.6	16 ± 2	70 ± 18

- stand for “no data”.
